# A luciferase based viability assay for ATP detection in 384-well format for high throughput whole cell screening of *Trypanosoma brucei brucei *bloodstream form strain 427

**DOI:** 10.1186/1756-3305-2-54

**Published:** 2009-11-12

**Authors:** Melissa L Sykes, Vicky M Avery

**Affiliations:** 1 Eskitis Institute for Cell and Molecular Therapies, Griffith University, Eskitis Building N27, Brisbane Innovation Park, Don Young Road, Nathan, Queensland, Australia

## Abstract

**Background:**

Human African Trypanosomiasis (HAT) is caused by two trypanosome species, *Trypanosoma brucei rhodesiense *and *Trypanosoma brucei gambiense*. Current drugs available for the treatment of HAT have significant issues related to toxicity, administration regimes with limited effectiveness across species and disease stages, thus there is a considerable need to find alternative drugs. A well recognised approach to identify new drug candidates is high throughput screening (HTS) of large compound library collections.

**Results:**

We describe here the development of a luciferase based viability assay in 384-well plate format suitable for HTS of *T.b.brucei*. The parameters that were explored to determine the final HTS assay conditions are described in detail and include DMSO tolerability, Z', diluents and cell inoculum density. Reference compound activities were determined for diminazene, staurosporine and pentamidine and compared to previously published IC_50 _data obtained. The assay has a comparable sensitivity to reference drugs and is more cost effective than the 96-well format currently reported for *T.b.brucei*.

**Conclusion:**

Due to the reproducibility and sensitivity of this assay it is recommended for potential HTS application. As it is commercially available this assay can also be utilised in many laboratories for both large and small scale screening.

## Background

Sleeping sickness, or Human African Tropanosomiasis (HAT), is a disease caused by two species of trypanosomes, *Trypanosoma brucei rhodesiense *and *Trypanosoma brucei gambiense*. The disease occurs in 36 countries in sub Saharan Africa. In 2006, there was an estimated cumulative rate of 50000-70000 cases worldwide [1]. The disease occurs in two stages, the first whereby trypanosomes multiply in the blood and lymphatic system, and includes symptoms such as headaches, fever, joints pain and itching. In the second stage, trypanosomes cross the blood brain barrier to the central nervous system and neurological symptoms often become apparent. Without treatment of the infection, the disease becomes fatal.

HAT chemotherapy relies upon a limited number of drugs, which are not effective against all stages or trypanosome species, have associated side effects or impractical administration regimes. Two of these drugs, suramin and pentamidine, are only effective against the first stage of disease, and are selective against *T.b.rhodesiense *and *T.b.gambiense*, respectively. Melarsoprol, which is effective against the second stage of the disease, causes encephalopathy in 5-10% of treated patients [2]. Eflornithine, also a second stage specific drug, is less toxic however is not effective against *T.b.rhodesiense *[3]. Suramin, pentamidine, melarsoprol and eflornithine all require intravenous injection for treatment which is not practical considering the poor medical facilities in most of the disease endemic areas. There also has been an increase in melarsoprol-refractory sleeping sickness cases which suggests possible emergence of melarsoprol-resistant trypanosomes [4,5].

The strong need for new, less toxic HAT drugs, which are particularly effective against the second stage of the disease has resulted in a variety of approaches to identify new treatments which are not uncommon approaches within anti-parasitic drug discovery. These options include combinations of existing HAT drugs (such as eflornithine and melarsoprol; [6]), new indications for existing drugs for unrelated disorders and improvements to a number of known drugs and compound classes [7]. High throughput screening (HTS) of whole parasites against large compound libraries has also been undertaken in the search for new therapeutic candidates [8]. The application of HTS in drug discovery has been explored in neglected disease area for some time, as this approach has the potential of identifying new drugs with novel modes of action. Thus, HTS remains an important consideration for HAT drug discovery programmes.

HTS of specific targets have been reported in the literature for *T.brucei *species. Assays in 384-well format have been developed to detect trypanothione reductase activity [9,10], however these do not involve the whole parasite. There have been few reports in the literature for HTS methods for whole cell screening for HAT. A viability assay which has been extensively reported in the literature for all *T. brucei *species and which utilises the dye Alamar Blue™ has been used a 96-well format by a number of authors [11-14]. We have recently developed a 384-well Alamar Blue assay for *T.b.brucei *whole cell viability estimation [15]. As an alternative to the Alamar Blue assay, a luciferase viability assay has recently been published for *T.b.brucei *whole cell screening in a 96-well format [16]. This paper reports the use of a bioluminescent ATP detection assay for HTS for a screening set of 2000 compounds. The same author has also reported the use of this assay for a smaller study of protease inhibitors against *T.b.brucei *[17]. It would be of benefit, in terms of time and cost considerations, to develop such a viability assay for *T.b.brucei *whole cells in a 384-well format for HTS. *T.b.brucei *has been routinely used in screening for initial identification of anti-trypanosomal compounds for potential anti-HAT drugs [7]. *T.b.brucei *causes trypanosomiasis in cattle [18] and is non-infective to humans [19]. For identification of lead compounds following HTS, actives are subsequently screened against human infective HAT strains for confirmation of activity.

The luciferase based viability assay described here utilizes firefly luciferase to detect intracellular ATP levels in viable cells. The reaction involves the mono-oxygenation of luciferin catalysed by luciferase in the presence of ATP, magnesium and molecular oxygen, the reaction yielding a luminescent signal. This assay has previously been used for determining viability of various cell types, and numerous kits are commercially available from a number of different suppliers. These kits, such as CellTiter- Glo^®^, are quick and efficient, with only 10 minutes required for signal development. In comparison, the Alamar Blue assay has a 6-24 hour incubation period for *T.b.brucei *cell viability assays reported in a 96-well format [5,11-13]. Although many published 96 well assay formats employ Alamar Blue development at 5% CO_2 _and 37°C inclusive of a total assay incubation time, we recently found these conditions to reduce the growth of *T.b.brucei *during 384 well plate assay optimisation [15]. We used a 24 hour incubation at room temperature with Alamar Blue following 72 hours of cell growth, for further signal development, as cell growth was reduced under these conditions (40 hours doubling time). We also found assay sensitivity was not significantly affected by this extra incubation. In this case, turn around would be faster for the luciferase assay as the development of signal is more rapid.

Here we report the development of a 384-well whole cell luciferase based viability assay for the detection of the viability of *T.b.brucei *whole cells. This assay was developed to HTS standards by incorporating the following statistical parameters: Z' factor (Z'), % coefficient of variation (% CV) and the signal to background assay window to reflect reproducibility. The Z' is a measurement of the reproducibility of an assay as described by Zhang and others [20]. This value is an indicator of the quality of an assay by analysing the variation of samples of positive and negative controls (no test compounds), and application of the use of this parameter to define the reproducibility of assays has been reported extensively [21]. An ideal assay would have a Z' of 1. A Z' of 0 would define an assay that could not be applied to screening due to poor reproducibility [22] and therefore would require further optimisation. The % CV denotes variation of an assay signal relative to the mean. It can be applied to one control variable rather than requiring both negative and positive controls. Generally, a Z' of greater than 0.5 with a % CV of less than 10 is acceptable for HTS. A % CV of greater than 17% indicates that the Z' will not reach 0.5 [23] and the closer to 0 the % CV is, the more reproducible, and therefore suitable an assay will be for HTS.

Optimisation of the established assay into 384-well format incorporated analysis of assay variables such as cell concentration, sensitivity to compound solvent, compound dilution medium and the IC_50 _values of a panel of reference compounds reported in the literature. The reference compounds used in this study consisted of the known drugs, pentamidine, and diminazene and a general kinase inhibitor, staurosporine. Diminazene and pentamidine have reported IC_50 _values from various publications which employ the Alamar Blue reagent for viability detection [11-13]. Diminazene is a registered veterinary drug used against *T.b.brucei*. Staurosporine has reported activity against *T.b.brucei *[16], as well as other trypanosome species, such as *Trypanosoma cruzi *[24] and *Leishmania *species [25].

The luciferase based ATP detection assay described here satisfies HTS assay criteria with a Z' of greater than 0.5. The assay is comparable with respect to known reference compound activities as those reported in the literature for the Alamar Blue assay in format [11-13], as well as the reported luciferase based assay in a 96-well format [16]. The parameters that were explored and optimised to provide a HTS assay in 384-well format are discussed in detail.

## Methods

### Parasites and in vitro culture

*T.b.brucei *bloodstream form strain 427 (BS427) was kindly supplied by Dr Achim Schnaufer, (Institute of Cell Biology, University of Edinburgh, UK) whilst at the Seattle Biomedical Research Institute (Seattle, WA, USA). The trypanosomes were maintained in log phase growth in 25 cm^2 ^tissue culture flasks (vented, 25 cm^2 ^Corning, Lowell, MA, USA) by sub culturing at either 24 or 48 hour intervals. Cells were grown in complete HMI-9 medium [26], supplemented with 10% FCS and 100 IU/mL penicillin/streptomycin (Invitrogen, Carlsbad, CA, USA). Cells were grown by incubating in conditions of 5% CO_2 _and 37°C in a humidified atmosphere.

### Statistical Analysis of Samples

For matrices of concentrations of cells and reagent, sample sets consisted of 8 wells. For initial set up experiments, combinations of conditions were analysed by comparing the signal to background ratio (cell concentrations versus addition of the same volume of HMI-9 medium alone).

For larger well samples, over one third of a 384-well plate, the signal to background ratio of control plates (1) and Z' (3) were calculated as per Zhang and colleagues [20].

Whereby

(1) S/B = mean signal/mean background

(2) SD = standard deviation

(3) Z' factor = 1- [(3*SD_A_+3SD_B_)/(meanA-meanB)]

where A = mean end point signal of each assay (cell growth detected in wells containing *T.b.brucei *in HMI-9 medium in a 384-well plate after 72 hours growth); B = background signal, HMI-9 media incubated for 72 hours with no cell addition, or with addition of 2.8 μM final pentamidine concentration.

For all reference drug compound IC_50 _estimations, activity at each compound dose was averaged from triplicate experiments and expressed ± SD in nM.

### Final optimised HTS assay conditions

The final optimised assay protocol, suitable for HTS, was established through variation of the conditions used as outlined. Unless stated otherwise, the basic HTS method was used. All additions of reagents and cells were made with a Multidrop™ liquid handler (Thermo Scientific, Newington, NH) under sterile conditions. Fifty-five μL of 500 cells/mL of *T.b.brucei *in HMI-9 medium were added to a white solid lidded 384-well plate (PerkinElmer, Waltham, MA, USA). Cells were incubated for 24 hours at 37°C with 5% CO_2 _in humidified conditions before addition of 5 μL of compound prepared in 100% DMSO, or 100% DMSO only for control wells. Compounds and/or DMSO were pre-diluted 1:40 in HMI-9 medium using a MiniTrak™ robotic liquid handler (PerkinElmer, Waltham, MA, USA). Five μL of diluted sample was added to the each well using the MiniTrak, giving a final DMSO concentration of 0.208%. Cells were incubated for a further 48 hours at 37°C, resulting in a final incubation time of 72 hours, of which trypanosomes were exposed to test compounds for 48 hours. Fifteen μL of a luciferase based reagent, CellTiter-Glo (Promega Inc, Madison, WI, USA) was then added for detection of intracellular ATP. The plate was shaken for 2 minutes to facilitate cell lysis and the release of intracellular ATP, and then incubated for a further 10 minutes at room temperature. The plate was read on a TriLux plate reader (PerkinElmer, Waltham, MA, USA), utilising a luminescence protocol.

### Luciferase assay and detection of exogenous ATP

To establish the limits of detection for ATP, varying concentrations of ATP from 8 nM to 400 μM (in HMI-9 medium) were added to a 384-well plate (as outlined in final assay conditions). Detection was performed by addition of 15 μL of the luciferase based reagent, as a mid-range volume trialed for this assay (volumes of trypanosomes were varied from 25 μL to 10 μL for initial growth experiments). Fifty-five μL of each concentration of the ATP solution were added to four 384-wells, with detection as outlined in final assay conditions.

### Doubling time of *T.b.brucei *in flask culture

The doubling time for *T.b.brucei *was originally calculated to be 6.8 hours in a flask culture [15]. To determine if this was constant over multiple *T.b.brucei *cell splits, the doubling time was calculated at 24 hour intervals by maintaining the final concentration of cells at 1 × 10^6^cells/mL and the cells were estimated to be in log phase over this time period. The calculation was based upon manipulation of the doubling time equation, using a known doubling time of 6.8 hours.

To determine if the doubling time was stable at 6.8 hours, the prepared culture dilution was signified as quantity (q_1_) at time (t_1_) and the resultant density following 24 hours incubation was quantity (q_2_) at time (t_2_) as per the doubling time equation:

Where T_d _= doubling time.

To compare the doubling time of a flask culture to that of a culture in a well, the same cell density of trypanosomes in 55 μL was added to wells of a white solid lidded 384-well plate as to a flask culture, to gain an estimated 1 × 10^6 ^cells/mL following 24 hours growth. Three wells from the plate and 3 replicates from the flask were counted and compared for doubling time.

### Detection of trypanosomes inoculated from flask grown cultures in to a 384-well plate

Fifty-five μL of trypanosomes at 3 × 10^6^cells/mL were diluted to 1 and 2 × 10^6^cells/mL, respectively and transferred directly to a 384-well plate. Twenty-five, 20, 15 and 10 μL of CellTiter-Glo reagent were added to the replicate volumes of trypanosomes. Detection was as described in the final assay conditions, with the only variation being the reagent volume. The % CV and signal to background ratio of well samples was used to define ideal reagent volumes to employ for further assay set up for growth in 384-well plates.

To further estimate the limitations of detection for the ATP detection assay with respect to cell concentration, 55 μL of varying densities of trypanosomes in a larger range of doses from 976 cells/mL to 3 × 10^6^cells/mL in HMI-9 medium were added to a 384-well plate. Cultures diluted from were at 3 × 10^6 ^cells/mL (for above 1 × 10^6 ^cells/mL density); and 1 × 10^6 ^cells/mL. Detection was undertaken as described in the final assay conditions.

### Growth and detection of trypanosomes in 384-well plate

Plates were inoculated at a variety of cell concentrations from 0 to 4000 trypanosomes/mL in 55 μL of HMI-9 medium and incubated at 37°C in 5% CO_2 _for 72 hours. Three wells were harvested and counted with a haemocytometer at each 24 hour period to estimate growth. CellTiter-Glo reagent was added to each cell concentration at each time interval at either 10 or 15 μL additions for comparison. The signal to background ratio and % CV of sample wells for each assay and trypanosome/reagent volume combinations were estimated. To obtain statistically relevant data each treatment was performed over 8 wells of replicates.

### Reference Compound IC_50 _determination

The IC_50 _value of each reference compound was calculated by plotting % inhibition (100% inhibition was equal to 2.8 μM final pentamidine) against log [reference compound] in the software package PRISM 4 graph pad (denoted IC_50 _by PRISM). The IC_50 _value was the concentration of compound that was estimated to lead to 50% of growth; with the minimum of 0% growth (cells co-incubated with 2.8 μM pentamidine) and 100% growth (no compound addition). Each dose was screened in triplicate.

### Optimisation of DMSO concentration and compound dilution medium

Fifty-five μL of *T.b.brucei *at a density of 500 cells/mL was inoculated into a 384-well plate and incubated for 24 hours. Five μL of varying concentrations of DMSO diluted in HMI-9 medium with no FCS were added to each plate. Plates were incubated for a further 48 hours and the HTS protocol, as outlined in the final assay conditions, was undertaken. One quarter of a 384-well plate was used to calculate the Z' for each treatment.

The final assay conditions for the HTS protocol as outlined in the methods were used to estimate the IC_50 _values of the reference compounds pentamidine, diminazene and staurosporine in three independent experiments. The final optimised conditions included an inoculum of 500 cells/mL, 15 μL of CellTiter-Glo and a final DMSO concentration of 0.208%, with no FCS in the dilution medium.

For optimisation of compound dilution medium, additions of HMI-9 medium, water, HMI-9 medium without FCS supplement and PBS were compared with respect to assay sensitivity to reference compounds. The final assay conditions used in this experiment were varied only by the diluent used.

## Results

### Luciferase assay signal from exogenous ATP

**Figure 1:**
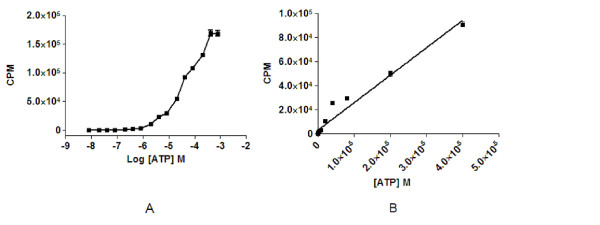
**Detection of exogenous ATP with the luciferase assay**. (A) Fifty-five μL of ATP in HMI-9 medium detected by 15 μL of CellTiter-Glo reagent over a dose range resulting in a plateau of the signal at 400 μM. (B) Representation of a linear range of ATP detection with CellTiter-Glo over varying concentrations of ATP. Linearity was approximately 40 μM before the signal plateau. Each concentration was screened in triplicate. The experiment was repeated twice.

The luciferase assay could detect ATP concentrations with a signal window (to background of HMI-9 medium with no ATP addition) down to the lowest dose tested at a final concentration of 8 nM, with a 15 μL addition of detection reagent to 55 μL of ATP solution in HMI-9 medium. The signal window was 24.5 ± 2.6 times that of media with no ATP, with a mean signal of 90.4 ± 9.4 CPM for 8 nM of ATP. The highest concentration that could be detected within the linear range was 40 μM ATP with a signal window estimated to be 2.9 × 10^4 ^± 1439 times with a luminescent signal of 9.1 × 10^4 ^± 3504 CPM (Figure 1A). There was a linear relationship to this point, with an r^2 ^estimated at 0.96 (Figure 1B). The signal had begun to plateau at 400 μM, with a mean value of 1.70 × 10^5 ^± 5600 CPM (Figure 1A).

### Doubling time of *T.b.brucei *in flask culture

The doubling time in a flask of *T.b.brucei *cultures of cells did not vary from the estimate previously made at 6.8 hours. We did not find a significant variation in a flask versus plate culture doubling time with a ratio of 0.97 ± 0.05 over 3 replicate independent experiments.

### Detection of trypanosomes from a flask grown culture transferred directly to a 384-well plate

**Figure 2:**
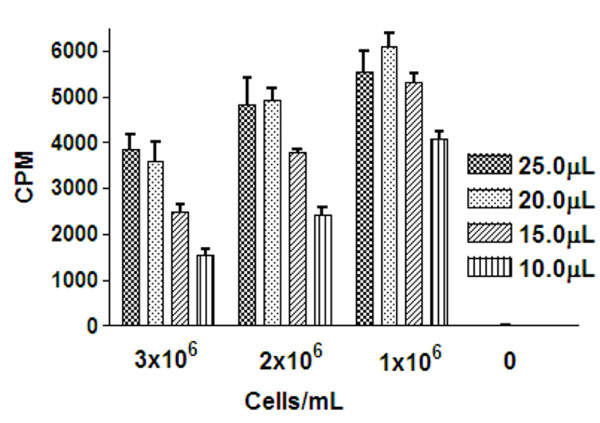
**Combination of cell concentrations of *T.b.brucei *and reagent volumes for signal detection when a 384-well plate inoculated directly from flask culture dilutions and detected by the luciferase ATP detection assay**. Fifty-five μL of *T.b.brucei *cell densities ranging from 1-3 × 10^6 ^cells/mL were transferred from a flask culture in to a 384 well plate and detected with CellTiter-Glo. Twenty-five, 20, 15 and 10 μL volumes of CellTiter-Glo were compared for signal detection and reproducibility.

A decrease in the cell density of trypanosomes from 3 to 1 × 10^6 ^cells/mL, for all of the luciferase reagent volumes tested, resulted in an increase in the mean CPM, with the highest counts seen at 1 × 10^6^cells/mL (Figure 2). Detection with 25 μL of luciferase reagent resulted in the greatest signal window across treatments; with a maximum of a 657 times window of cells to HMI-9 medium with no cell addition, and a % CV of 8.8%. A reduction to 20 μL reduced the % CV to 5.2% and to 15 μL the % CV was 3.7%. The signal window for 15 uL of reagent was 646 times. In consideration of cell growth likely resulting in higher well to well variation compared with direct inoculation in the plate, the lowest % CV with the highest signal window at 1 × 10^6 ^cells/mL was used as a criteria for the concentration and volume used for further investigation, which was with addition of 15 μL of the CellTiter-Glo reagent.

**Figure 3:**
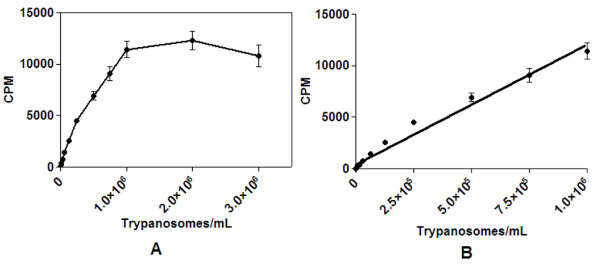
***T.b.brucei *cells directly transferred from a flask culture to a 384 well plate and ATP detected with 15 μL luciferase based reagent**. (A) Cell concentrations of *T.b.brucei *and detection by the luciferase based assay of 55 μL per well of over a dose range to 3 × 10^6 ^cells/mL with cells transferred directly from dilutions made from flask grown cultures. 15 μL of luciferase reagent was added for detection. The mean CPM was estimated over 4 replicates from two separate experiments. (B) There was a linear relationship between cell number and trypanosomes to 1 × 10^6 ^cells/mL (r_2 _= 0.99).

Figure 3A shows that the trend of a reduction in CPM seen in initial experiments from densities of 1-3 × 10^6^cells/mL was replicated with a 15 μL detection volume of luciferase and 55 μL of trypanosomes, with intermediate doses. Cell concentrations were at 3 to 0.75 × 10^6 ^cells/mL then in 0.5× increments from 0.5 × 10^6 ^cells/mL to 488 cells/mL. Detection of 3 × 10^6 ^cells/mL of trypanosomes resulted in a mean CPM of 1.1 × 10^4 ^± 1046, with a CV of 9.6%. A reduction of the cell density to 1 × 10^6 ^cells/mL resulted in an increased signal, with a reduced % CV, to 7.5%. Detection of 2 × 10^6 ^cells/mL resulted in a mean CPM of 1.2 × 10^4 ^± 911 and CV of 8.9%; however the signal had plateaued from 1 × 10^6 ^cells/mL. The greatest signal window closest to the linear end of detection was therefore observed to be at a 1 × 10^6 ^cells/mL density (Figure 3B).

### Growth and detection of trypanosomes in 384-well plate

**Figure 4:**
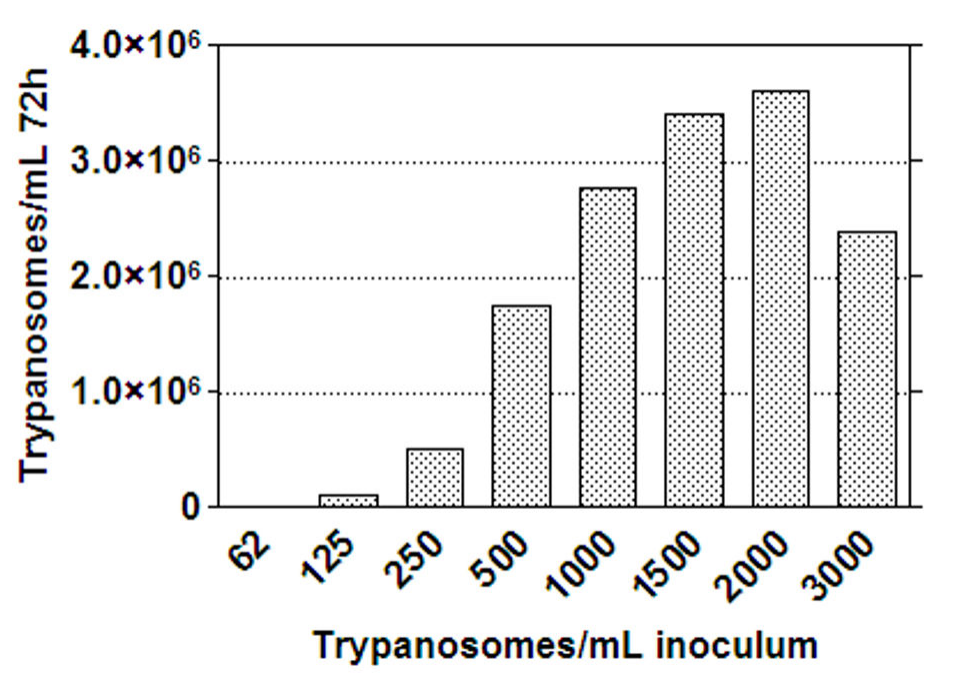
**Cell counts of *T.b.brucei *following growth at 72 hours in a 384-well plate**. Varying inocula of trypanosomes were grown in 55 μL of HMI-9 media and harvested for counting after 72 hours growth.

**Figure 5:**
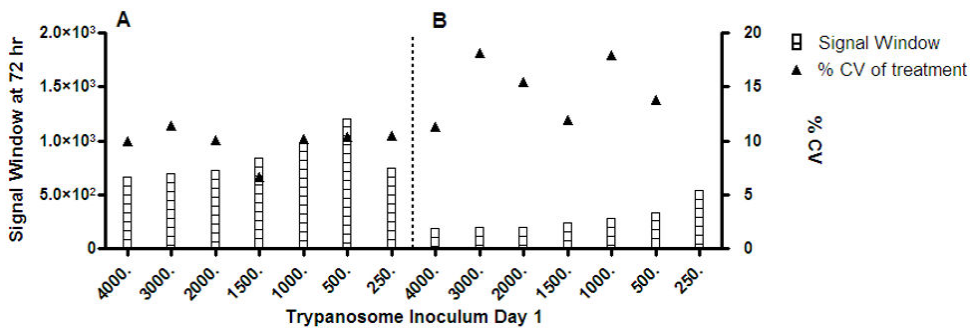
**Signal window and % CV of samples from varying inocula of trypanosomes following 72 hours growth and detection with the luciferase viability assay**. The assay signal window and % CV after 72 hours growth with differing trypanosome inocula were compared with both 15 μL (A) and 10 μL (B) of detectant. The signal window was calculated from media without a cell addition.

Initial studies indicated that 1 × 10^6 ^cells/mL, with 15 μL of detectant, provided an optimum signal to background ratio for the assay. To determine the most suitable inoculum density to obtain an optimal parasite growth in a 384-well plate, whilst taking into consideration assay performance over a 72 hour period, the cell number used as the inoculum per well was titrated as follows 0, 62, 125, 250, 500, 1000, 1500 and 2000 cells/mL. Growth by cell count estimations of cells following 72 hours incubation are shown in Figure 4. Approximately 3 × 10^6^cells/mL was the maximum attainable number using an inoculum of 2000 cells/mL. An increase in inoculum to 3000 cells/mL resulted in a decrease in cell number. The signals obtained for differing cell concentrations, with 10 and 15 μL of luciferase reagent are shown in Figure 5. The greatest assay signal to background window was obtained when 15 μL of the luciferase reagent was used for detection of trypanosomes. With a 10 μL detection reagent addition across cell concentrations a reduced signal was observed. The lowest % CV and greatest window combination was observed using an inoculum of 500 cells/mL. This inoculum produced 1.7 × 10^6 ^cells/mL (Figure 4). A slight decrease in CPM was seen from 1000 cells/mL inoculum (a final density of 2.7 × 10^6 ^cells/mL) and a plateau of the signal from 2000 cells/mL inoculum (a final cell density of over 3 × 10^6 ^cells/mL).

### Reference compound IC_50 _estimation and cell inoculum

Reference compounds (outlined in methods) were screened over a dose range of cell inoculum densities of 2000, 1500, 1000, 500 and 250 cells/mL and estimations of reference compound IC_50 _values were initially undertaken by the serial dilution of compound stocks in 100% DMSO in to HMI-9 medium (Table 1). A 5 μL volume of these dilutions was added to each well, following an initial 24 hours incubation of cells in the absence of the compound, which was to determine cell growth in the plate, and to allow the equilibration of growth of the parasite in the plate. The DMSO concentration used was at a final concentration of 0.27% at the top dose in the assay for diminazene, 0.08% for pentamidine and 0.20% for staurosporine, DMSO is less likely to significantly affect the assay at these low concentrations. The next serial dilution was at a third of this concentration.

**Table 1:** Luciferase assay estimated activities over varying *T.b.brucei *inocula for the reference compounds staurosporine and diminazene.

Cell density/mL	Staurosporine	Diminazene
**2000**	13.50 ± 8.5	453.8 ± 130.2
**1500**	19.90 ± 1.3	138.1 ± 4.4
**1000**	8.73 ± 3.2	178.2 ± 57.7
**500**	7.60 ± 0.6	123.4 ± 6.4
**250**	7.96 ± 1.2	61.80 ± 7.1

IC_50_values for each reference compound/cell inoculum concentration are expressed in ± standard deviation, in nM.

Reference IC_50 _values were compared to those previously published from 96-well methodologies for the Alamar Blue assay [11-13] and a 96-well methodology for the luciferase assay [16]. A 500 cells/mL inoculum resulted in an IC_50 _value of 123.4 nM for diminazene, 7.6 nM for staurosporine (Table 1) and 3.6 nM for pentamidine (results not shown). A reduction in the cell inoculum to 250 cells/mL reduced the IC_50 _value of compounds slightly; diminazene 2 times and pentamidine 1.4 times, however the staurosporine IC_50 _value was not affected. Increasing the cell number resulted in a decrease in sensitivity for all compounds, and an increase in the variability of replicates.

### Optimisation of DMSO concentration

**Figure 6:**
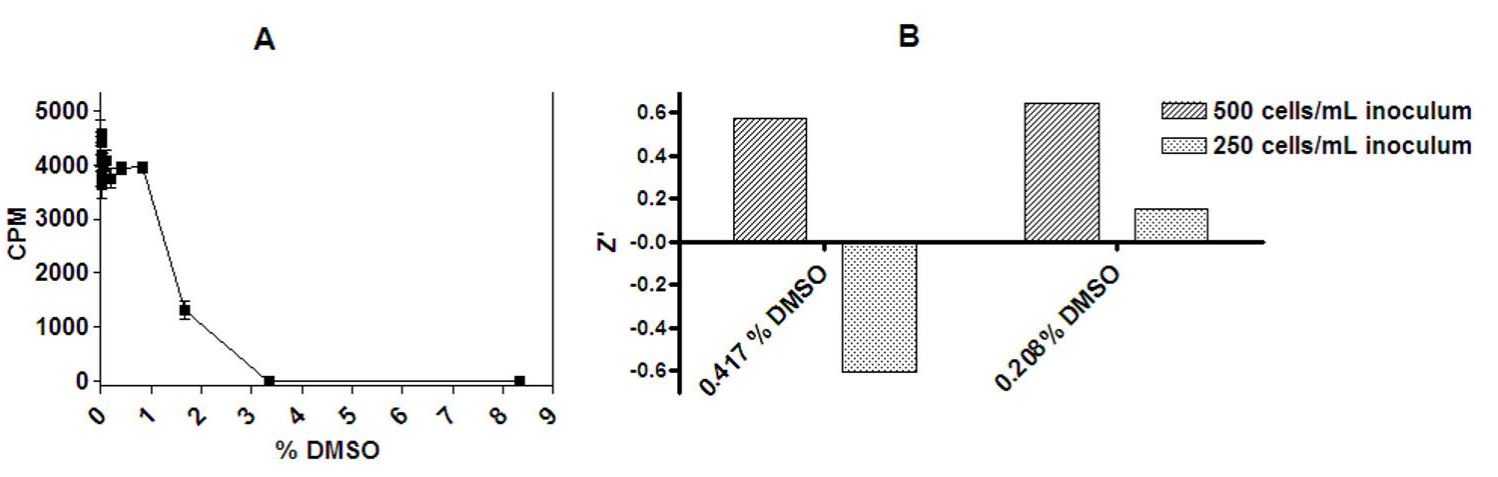
**DMSO dose response curve for 500 cells/mL inoculum; and the Z' of pre-diluted DMSO at 0.417% and 0.208% final concentration in the assay for a 500 and 250 cells/mL inoculum density**. (A) DMSO standard curve from 0 to 8.3% DMSO in the luciferase assay for a 500 cell/mL inoculum (B) DMSO concentration and Z' of 500 and 250 mL cell inocula with a pre-dilution of DMSO to decrease the assay concentration. The Z' was estimated from a quarter of a 384 well plate.

Final DMSO concentrations from 0 to 8.3% DMSO were initially tested for a 500 cells/mL inoculum and it was found there was a decrease in the assay signal exhibited from greater than 1% DMSO in the assay (Figure 6A). It was found that using a final concentration of 0.5% DMSO in the assay, however, resulted in a poor reproducibility across the plate, with a Z' of 0.45 (results not shown). Experiments were undertaken with a pre-dilution of DMSO before addition to the plate, using a 1:20 and 1:40 dilution before the addition of 5 μL of this dilution volume to the assay volume (Figure 6B). The resulting final DMSO concentrations were 0.417% and 0.208%, respectively Results were replicated for both 250 and 500 cells/mL inocula. The Z' for detection using a final DMSO concentration of 0.417% and a 500 cells/mL inoculum was 0.57. Decreasing the cell inoculum to 250 cells/mL resulted in a Z' of -0.60%, reflecting poor reproducibility for this cell concentration.

For a final DMSO concentration of 0.208%, the Z' was increased to 0.65 (Figure 6B). For 250 cells/mL, a 0.208% DMSO concentration gave a Z' of 0.15 which consequently was not suitable for screening. The DMSO concentration may have to be diluted further to enable this cell concentration to be employed in HTS.

Comparisons were made between manual and automated additions of reagents for these dilutions. No difference in the Z' was observed between the manual and automated dispensing (results not shown).

### Optimisation of compound dilution medium

Reference compounds diluted in water resulted in a decrease in sensitivity when compared to results obtained following dilution of compounds with HMI-9 medium (Table 2). Dilutions made with HMI-9 medium without FCS, compared with FCS containing medium, had a negligible effect on the estimated IC_50 _values excepting the staurosporine IC_50_, increased slightly by 2.1 times. PBS increased the IC_50 _values with the exception of diminazene, by a factor of 1.8 and 2.1 times for pentamidine and staurosporine respectively, in comparison to medium with no FCS.

**Table 2:** Reference compound IC_50_'s from reference compounds diluted in water, HMI-9 medium with and without FCS and PBS.

Compound	PBS	Water	FCS	No FCS
**Pentamidine**	13.42 ± 7.5	57.78 ± 40	9.23 ± 0.71	7.56 ± 2.8
**Diminazine**	65.98 ± 1.7	140.6 ± 79	65.79 ± 21	62.46 ± 21.3
**Staurosporine**	10.99 ± 1.3	12.27 ± 3.0	2.43 ± 0.59	5.22 ± 1.1

IC_50 _values of compounds in variations of compound dilution media were determined in the luciferase assay and calculated from duplicate samples, expressed as ± SD, in nM.

The mean IC_50 _values of reference compounds (n = 3) were estimated using the final HTS conditions. Staurosporine had an IC_50 _value of 1.94 ± 0.09 nM, diminazene 60.88 ± 27.69 nM and pentamidine 11.91 ± 4.86 nM.

## Discussion

A number of assay formats have been used to screen trypanosome species including ^3^H-hypoxanthine incorporation, nuclear staining techniques, flow cytofluorometry and measurement of culture pH [27]. The Alamar Blue assay was developed to improve associated assay variables such as long incubations, costly reagents and poor assay reproducibility/signal [14] and the assay has been extensively reported in the literature [5,11-14]. More recently, a luminescence assay to detect intracellular ATP has been used in 96-well format [16,17]. Such an assay could be an alternative assay for HTS to the commonly used Alamar Blue assay. When comparing the feasibility of using these assays one point is the necessity to add Alamar Blue under sterile conditions, which may impact on potential robotic manipulations. Some HTS robotic systems may not be under sterile conditions and therefore the luciferase assay may be favoured to decrease manual work associated with the end point addition. However, the luciferase assay requires plate shaking for cell lysis and not all HTS systems are capable of this step. Both assays are, however, commercially available and this allows access to these reagents for many laboratories for comparative development, dependant upon the capability of the laboratory equipment at hand.

We have described a luciferase based ATP detection assay for estimation of *T.b.brucei *viability in a 384-well plate for a detection of a small volume of trypanosomes (55 μL) compared with previously employed 96-well protocols which utilised a sample volume of 100 μL [16,17]. The assay described here is reproducible, conforms to HTS standards with a Z' of 0.65 (Figure 6B), and has similar IC_50 _values for known *T.b.brucei *reference compounds as previously reported Alamar Blue assays, a luminescent viability assay (Table 3) and also to a recently reported Alamar Blue assay in 384-well format [15].

**Table 3:** Reported activities for *T.b.brucei *reference compounds.

Compound	Reference	IC_50 _(nM)	Assay
**Diminazene**	[11]	90	Alamar Blue
**Diminazene**	[12]	500	Alamar Blue
**Diminazene**	[13]	990	Alamar Blue
**Pentamidine**	[11]	10	Alamar Blue
**Pentamidine**	[13]	40	Alamar Blue
**Staurosporine**	[16]	7	Luciferase

IC_50 _values from various publications are listed for comparison. Assay formats include variations of the Alamar Blue viability assay and ATP detection using bioluminescence.

Variables that were explored to determine the best conditions to use in the 384-well assay included cell concentration, volume of detection agent, DMSO sensitivity and the compound diluent. The concentration of the cell inoculum to provide optimal cell growth and detection under our experimental conditions was found to be 500 cells/mL. For cell growth, an inoculum of 2000 cells/mL produced a maximum cell number of 3 × 10^6 ^cells/mL following 72 hours incubation. In comparison, an inoculum of 3000 cells/mL resulted in a decrease in the final cell number (Figure 4). This would be most likely attributed to the fact that the culture is in decline, with the occurrence of observed cell death. We have previously seen this decline in cultures exceeding over an approximated density of 3 × 10^6 ^cells/mL [15]. This density maximum density had also previously been determined from other studies for other *T.brucei *bloodstream species to be 3 × 10^6^cells/mL [28]. The doubling time reported here was calculated to be on average 6.8 hours which is also similar to previously reported doubling times for other *T. brucei *bloodstream species in HMI-9 medium [28].

A cell inoculum of 2000 cells/mL produced the maximum cell number (Figure 4, just over 3 × 10^6 ^cells/mL) and this cell concentration was synonymous with a slight decrease in the luciferase signal in comparison to 1 or 2 × 10^6 ^cells/mL with cells directly transferred from a flask (Figures 2 and 3). The signal was also reduced in comparison to cell inocula of 1000 and 500 cells/mL grown in a plate (Figure 5). As cell number was at maximum from cells grown from an inoculum of 2000 cells/mL, this signal decrease could be a function of cells reaching a stationary phase of growth and therefore the ATP production decreasing. This phase related decline in ATP production has been observed in other cell lines [29-31] and is synonymous with a high cell density in fibroblast cell culture [32]. Cell density has been shown to regulate trypanosome number in infections by differentiation induced by stumpy induction factor at high concentrations [33], and it could be suggested that other metabolites may be produced at higher cell densities which effect cultures in monomorphic forms. As cells detected directly from a flask culture exhibited a decrease in CPM at 3 × 10^6 ^cells/mL in comparison to 2 × 10^6 ^cells/mL (diluted from a 3 × 10^6 ^cells/mL culture density; Figure 2), there may have also been interference from ATPases released from the maximum concentration of lysed cells, reducing the signal. This signal interference was not a function of the maximum ATP that could be detected in the well, as this signal plateaued at much higher CPM (Figure 1).

Titrating down to lower densities of trypanosomes revealed a linear relationship between cell number and signal between 1 × 10^3 ^to 1 × 10^6 ^cells/mL trypanosomes from cells directly transferred from a flask grown culture (Figure 3B). Mackey and colleagues [16] found a linear relationship between trypanosome concentration and luciferase signal to 6 × 10^4 ^cells/well. As the inoculum for this assay was 1 × 10^5 ^cells/mL, with a doubling time estimated at 6.8 hours for example, the final assay concentration of cells following 48 hours incubation would have reached over maximum, at 3 × 10^6 ^cells/mL, or 3 × 10^5 ^cells/well, so it can not be determined if a similar effect was seen in this assay with the concentrations examined for a linear trend.

With plate inoculated cultures, the cell number that produced the greatest signal window was 500 cells/mL (Figure 5). This inoculum resulted in approximately 1.7 × 10^6 ^cells/mL following 72 hours growth in plate (Figure 4). This suggested a slightly faster doubling time in the plate, as forecast by the doubling time equation this cell inoculum should be under 1 × 10^6 ^cells/mL. However, at this low cell inoculum, there is not a linear trend in growth as we have previously observed [15], and therefore the doubling time would not be expected to be constant. Relating this concentration to the linearity of cell number versus CPM from direct cell transfer, suggested that this cell concentration was slightly above the linear range of the assay, which was at a maximum of 1 × 10^6 ^cells/mL (Figure 3B). Taking this into consideration, it was determined that reduction of the cell number to less than 1 × 10^6 ^cells/mL final in the assay, or an inoculum of 250 cells/mL of trypanosomes, could be applied for HTS. The DMSO tolerability for 250 compared with 500 cells/mL was explored at both a 0.417% and 0.208% final DMSO concentration. The assay incorporating a 500 cells/mL inoculum at 0.208% DMSO resulted in the least variability, reflected in a Z' of 0.64 (Figure 6B). However, a 250 cells/mL inoculum resulted in a Z' of less than 0.5 for both DMSO concentrations and therefore was not acceptable for HTS application. The DMSO concentration would need to be further reduced to allow this inoculum to be applied, which would bring about potential library concerns as samples would have to be diluted further before screening, therefore potentially reducing the sensitivity of the assay at this cell density. Mackey and colleagues [16] employed a DMSO concentration of 1% at the stage of cell addition in a 96-well luciferase assay. The 384-well assay would have an estimated cell number at the time of addition of DMSO of around 345 cells/well (estimating a doubling time of 6.8 hours) in comparison to an estimated 1 × 10^4 ^cells/well in the 96-well format which could explain the differences in the necessity for a lower DMSO requirement for 384-well screening using the HTS conditions as outlined here.

Table 3 shows reference compound activities for both reported Alamar Blue assays and a reported luciferase based ATP detection assay in 96-well format. We found that a 500 cell/mL inoculum resulted in IC_50 _concentrations that were comparable to those found in the literature from dosing compounds with differing trypanosome inocula. From Table 1, increasing the cell density decreased the sensitivity to both diminazene and staurosporine, when making comparisons of inoculums of 500 cells/mL with 2000 cells/mL. There was a 3.7 and 1.8 times reduction in the IC_50_values of diminazene and staurosporine, respectively. A 3.3 times reduction in activity was also revealed for pentamidine (results not shown). It is not possible to directly compare the Alamar Blue assay formats to the assay reported here due to the detection reagents used, incubation time, media differences and cell inoculation variables between assays. However, a similar sensitivity was observed with results obtained by Lanteri and collegues [11], with an IC_50 _value for pentamidine of 10 nM and diminazene 90 nM reported, compared to those observed, pentamidine 11.91 nM and diminazene 60.88 nM. The sensitivity to staurosporine observed using a 500 cells/mL inoculum (an IC_50 _of 1.94 nM) was comparable to the 96-well format previously reported [16] (IC_50 _of 7 nM). As there may have been some interference with a higher cell density used by these authors in comparison to the protocol reported here, and as staurosporine has a high Hill Slope (> 3 over all cell doses), further experiments would further determine sensitivities between these assay formats with other known anti-trypanosomals.

Importantly, the assay inoculum employed in the HTS assay developed fits HTS statistical criteria The assay is within the linear range of the detection of the reader (Figure 1), and approximately 0.7 × 10^6 ^cells/mL from the linear range of detection of cells estimated in the assay system (Figure 3B). The assay at the defined cell inoculum of 500 cells/mL has sensitivity to known compounds similar to published reference compound activity in other assay formats (Table 3).

The potential diluent used for the addition of compounds and/or controls to the assay was explored, as pre-dilution of DMSO, due to DMSO sensitivity, was necessary. During a HTS campaign it is not uncommon for the screening library to be pre-diluted into the assay plate prior to HTS commencing. Exclusion of FCS from such a dilution medium was considered as this may reduce the risk of certain contaminating microorganisms [34], considering that there is a brief exposure of plates to the atmosphere whilst the dilution is carried out. FCS has also been found to influence cellular toxicity due to components binding to inhibitor compounds [35] and therefore dilutions and storage in a medium with FCS may potentially influence compound activity. Thus, FCS was not included in the diluents used here. The use of water as a diluent increased the IC_50 _value of the reference compounds by 7.6, 2.3 and 2.4 times, respectively for pentamidine, diminazene and staurosporine in comparison to medium without FCS (Table 2). There was also a decrease in the cell number by 1.5 times with the water addition (results not shown). This could have been as a result of a slight difference in the buffering capacity of the system or an osmotic effect of water on the cells, potentially causing cell lysis. For the optimal 384-well assay performance, HMI-9 medium with exclusion of the FCS component was the diluent of choice, as it did not significantly change the IC_50 _value of the reference compounds.

## Conclusion

We have reported here the successful development and optimisation of a luciferase based cell viability assay in 384-well format for use in HTS of *T.b.brucei *BS427, which exhibits benefits compared with previously published assay methodologies. Notably the assay is reproducible, fits statistical parameters, with a similar sensitivity to known drugs in comparison with previously published results and is cheaper than the currently used 96-well assay format for HTS. The assay described here is approximately three times less expensive per well than the 96-well luciferase assay currently reported in the literature. Importantly, the assay is biologically relevant, and has been shown statistically to perform well, adhering to the Z' criteria imposed (cut off of 0.5, but closest to 1 as possible). Additions of assay reagents and cells were made with automated liquid handlers, allowing for rapid additions for HTS and handling of a large plate numbers.

To our knowledge this is the first report of a 384-well cell viability assay for *T.b.brucei *utilising cellular ATP estimation, with a luciferase based detection agent. A future considerations is the comparison of the luciferase based 384-well assay to the Alamar Blue based 384 well assay [15] for HTS application with a larger panel of compounds.

## Competing interests

The authors declare that they have no competing interests.

## Authors' contributions

MLS carried out the experimental work and drafted the manuscript. MLS participated in the design of the study and performed data analysis. VMA conceived the study, participated in its design and coordination and contributed to the manuscript preparation. All authors read and approved the final manuscript.
